# Field‐Effect Transistors Based on 2D Organic Semiconductors Developed by a Hybrid Deposition Method

**DOI:** 10.1002/advs.201900775

**Published:** 2019-08-01

**Authors:** Zhiwen Zhou, Qisheng Wu, Sijia Wang, Yu‐Ting Huang, Hua Guo, Shien‐Ping Feng, Paddy Kwok Leung Chan

**Affiliations:** ^1^ Department of Mechanical Engineering The University of Hong Kong Pok Fu Lam Road Hong Kong; ^2^ Department of Chemistry and Chemical Biology University of New Mexico Albuquerque NM 87131 USA

**Keywords:** 2D, field‐effect transistors, monolayers, organic crystals, templates

## Abstract

Solution‐processed 2D organic semiconductors (OSCs) have drawn considerable attention because of their novel applications from flexible optoelectronics to biosensors. However, obtaining well‐oriented sheets of 2D organic materials with low defect density still poses a challenge. Here, a highly crystallized 2,9‐didecyldinaphtho[2,3‐b:2′,3′‐f]thieno[3,2‐b]thiophene (C_10_‐DNTT) monolayer crystal with large‐area uniformity is obtained by an ultraslow shearing (USS) method and its growth pattern shows a kinetic Wulff's construction supported by theoretical calculations of surface energies. The resulting seamless and highly crystalline monolayers are then used as templates for thermally depositing another C_10_‐DNTT ultrathin top‐up film. The organic thin films deposited by this hybrid approach show an interesting coherence structure with a copied molecular orientation of the templating crystal. The organic field‐effect transistors developed by these hybrid C_10_‐DNTT films exhibit improved carrier mobility of 14.7 cm^2^ V^−1^ s^−1^ as compared with 7.3 cm^2^ V^−1^ s^−1^ achieved by pure thermal evaporation (100% improvement) and 2.8 cm^2^ V^−1^ s^−1^ achieved by solution sheared monolayer C_10_‐DNTT. This work establishes a simple yet effective approach for fabricating high‐performance and low‐cost electronics on a large scale.

Since the debut of graphene, 2D materials have attracted much attention owing to their excellent mechanical, optical, thermal, and electrical properties.^1^, ^2^, ^3^, ^4^, ^5^, ^6^ Among these diverse 2D materials, 2D organic semiconductors as important contenders for practical organic electronics with perspective multifunctionality such as flexible displays,^7^, ^8^ integrated circuits,^9^, ^10^ and wearable sensors,^11^, ^12^ have been extensively studied. Comparing with their inorganic counterparts, organic semiconductors usually can be solution‐processed on a large scale with low cost, which enables the manufacture and commercial viability of electronic devices.^13^, ^14^, ^15^, ^16^, ^17^ Based on continuous efforts and investigations in recent years, a number of high mobility (over 10 cm^2^ V^−1^ s^−1^) organic field‐effect transistors (OFETs) based on semiconductors such as 3,11‐didecyldinaphtho[2,3‐d:2′,3′‐d′]benzo[1,2‐b:4,5‐b′]dithiophene (C_10_‐DNBDT),^18^ 3,11‐dioctyldinaphtho[2,3‐d:2′,3′‐d′]benzo[1,2‐b:4,5‐b′]dithiophene (C_8_‐DNBDT‐NW),^17^ and 2,7‐dioctyl[1]benzothieno[3,2‐b][1]benzothiophene (C_8_‐BTBT)^19^, ^20^ have been reported. Comparing with thermally evaporated organic thin films, 2D crystals have unique advantages of long‐range crystalline order, atomically flat surface with low defects, and low accessory resistance (resistance between the contacts and the channel) relative to non‐2D systems such as nanowires and thick crystals.^17^, ^20^ Therefore, intensive efforts have been devoted to developing large‐scale facile deposition techniques for the formation of highly crystalline ultrathin films. For instance, few‐layered C_8_‐BTBT molecular crystals grown by van der Waals epitaxy have demonstrated impressive electrical performance on foreign graphene or boron nitride substrates.^21^ Although the resulting C_8_‐BTBT showed a good crystal quality, 2D organic crystals fabricated by molecular beam epitaxy (MBE)^21^ and physical vapor transport (PVT)^22^, ^23^ have high energy costs because of the necessary ultrahigh vacuum processing environment and high deposition temperature. The demanding growth conditions limited their applications for the low‐cost and large‐scale processing of OFETs.

Some solution‐based techniques such as spin‐coating,^24^ dip‐coating,^25^ drop casting,^26^ inkjet printing,^27^, ^28^ screen printing,^29^ and floating coffee ring–driven assembly^20^ have been advanced to efficiently control the growth of highly crystalline films. However, it is still difficult to avoid morphological defects like voids and thermal cracks as well as small crystal domains with unaligned molecular packing, which limit the device performance and cause high device‐to‐device variation. The fast solvent evaporation and quick depositing speed in the above methods also result in polycrystalline thick films with a high density of grain boundaries because of stochastic and spontaneous nucleation. Furthermore, morphological cracking can appear at elevated temperatures due to anisotropic thermal expansion coefficients of semiconductors in different directions and/or between the semiconductors and dielectric layers.^30^, ^31^, ^32^ The induced structural transition under different temperature is related to the rearrangement of molecules and changes in intermolecular interactions and has a significant impact on charge transport properties.^31^, ^32^, ^33^ These grain‐boundaries and thermal‐crack defects provide the major trapping sites of charge carriers and cause device degradation under ambient air and moisture conditions.^34^, ^35^ Hence, their field‐effect mobility and device stability will be deteriorated. In addition to the morphological defects, the anisotropic nature of the molecular packing motif also causes variations in charge transportation.^36^ Qualitative correlation of the variation in charge carrier mobility with orientation has been reported for many organic semiconductors.^22^, ^37^, ^38^, ^39^, ^40^ To achieve high‐performance in real device integration, it is crucial to minimize or eliminate the variation in both crystal morphology and overall molecular arrangement. In this regard, finding ways to fabricate large‐area and ultrathin organic films with a preferential crystal orientation while significantly decreasing the morphological defects remains an essential challenge for growing 2D organic crystals.

Here, we report a novel hybrid deposition approach that combines the blade solution shearing method and common thermal evaporation technique for developing an ultrathin C_10_‐DNTT film with high crystallinity. These OFETs show superior performance to devices fabricated by either solution shearing or thermal evaporation. Specifically, we first fabricated a highly crystalline C_10_‐DNTT monolayer with large‐area uniformity by an ultraslow shearing (USS) method. The resulting crack‐free millimeter‐sized monolayer crystals were realized by intentionally locating the heterogeneous nucleation site and subsequently growing up in the orientation‐specific pattern at low temperature. By utilizing the 2D crystals as templates, another thin C_10_‐DNTT layer (10 nm) was then thermally deposited on the top of it. With the help of molecular interfacial‐interaction between the template and upper layer, the evaporated upper layer exhibits exactly the same crystal orientation as the template below. Inheriting monocrystalline merits from the solution shearing layer, the OFETs with hybrid C_10_‐DNTT films showed improved mobility of 14.7 cm^2^ V^−1^ s^−1^ averaged over a total of 45 devices with the highest mobility is 16.0 cm^2^ V^−1^ s^−1^. These devices are better than simple thermal evaporated or solution deposited devices, and their mobility is also on the high side of the reported C_10_‐DNTT values.

A monolayer, highly crystallized C_10_‐DNTT was grown by an ultraslow shearing method, as demonstrated in **Figure** **1**a. C_10_‐DNTT is a derivative of DNTT semiconductors which also has high mobility,^14^, ^41^ good thermal stability,^42^ and, more importantly, the solution processable capability. The meniscus‐guided blade shearing method offers excellent scalability and compatibility which makes it well suited for the fabrication of highly aligned 2D organic crystals. During the shearing process, the evaporation rate of the solvent at the edge of the meniscus line can be finely tuned by adjusting the temperature of the substrate and blade.^43^ Generally, the formation of a uniform film with a large domain size should satisfy two main requirements. First, heterogeneous nuclei need to be formed with a low density instead of random and simultaneous homogeneous nucleation. Second, the nucleation and kinetic growth processes should not occur simultaneously.^44^ According to classical nucleation theory, the supersaturation of the solution can stimulate the appearance of ordered clusters of atoms or molecules.^45^ These highly crystalline clusters, namely, nuclei, are likely to occur not only at the interface of the substrate and solution (heterogeneous nucleation) but also within the precursor solution (homogeneous nucleation).^44^ As shown in Figure 1c, the corresponding free energies ∆G*_Hom._ and ∆G*_Het._ are the maximum energy barrier that must be overcome to generate a mature crystal from solute molecules for homogeneous and heterogeneous nucleation, respectively. The heterogeneous nucleation is often favored due to its lower free energy barrier, which is proportional to the homogeneous nucleation free energy by a factor of *f*(θ). As Figure 1b illustrates, this factor is a function of the contact angle (θ) and given as^46^

(1) $$f\left(\theta \right)\;=\;\frac{1}{4}\left(2\;+\;\cos\theta \right){\left(1\;-\;\cos\theta \right)}^{2}$$

where cos θ = (γ_LS_−γ_NS_)/γ_LN_. Here, γ_LS_, γ_NS_, and γ_LN_ represent the surface energy of the liquid (L)‐substrate (S), nucleus (N)‐substrate (S), and liquid (L)‐nucleus (N) interfaces, respectively. For a higher energy surface with a lower contact angle, heterogeneous nucleation would thus be preferred.

**Figure 1:**
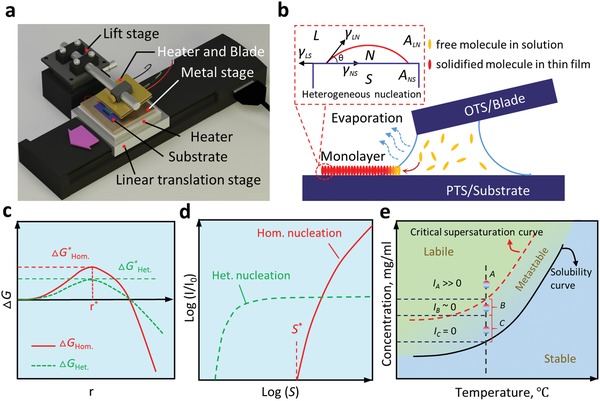
Ultraslow shearing (USS) method and classical nucleation/growth theories. a) Schematic diagram of the USS method. b) Schematic illustration of the growth of highly crystalline monolayer crystals by the USS method. c) The total Gibbs free energy change (∆*G*) relate to the radius (*r*) of the nucleus. Besides, r* indicates the critical nucleus radius and ∆G*_Hom._ and ∆G*_Het._ represent the maximum total Gibbs free energy change in homogeneous and heterogeneous nucleation process, respectively. d) Relationships between the normalized nucleation rate and supersaturation ratio (*S*) during homogeneous and heterogeneous nucleation mechanisms. e) Schematic, not to scale, representation of the critical supersaturation and stable saturation concentration of C_10_‐DNTT dissolved in an organic solvent as a function of the solution temperature.

Based on the classical nucleation theories, to enable the distinction of homogeneous and heterogeneous nucleation, the dependence of nucleation rate (*I*) to supersaturation ratio (*S*) is crucial.^44^, ^47^ Nucleation rate is the number of stable nuclei which exist per unit volume of the solution per unit time and supersaturation ratio is defined as the ratio between the solute concentration (*C*) and the solubility limit (*C_S_*), that is *S = C/C_S_*.^44^, ^47^ As schematically shown in Figure 1d, it is obvious that heterogeneous nucleation is only preferred at very low *S*. However, measuring the transient supersaturation ratio is difficult and complex. For the surface with fixed surface energy, one can simply control the supersaturation ratio by changing the solution concentration, since the evaporation rate is almost independent of the initial concentration.^48^ Hence, for the growth of uniform and high‐quality films with a large area, we intentionally promoted the solution condition to favor heterogeneous nucleation by precisely controlling of the solute concentration (*C*) and the deposition temperature (*T*). Meanwhile, the heavily doped silicon with 300 nm SiO_2_ was treated by trichloro(phenethyl)silane (PTS), a commonly used self‐assemble monolayer (SAM) to increase the substrate surface energy (Figure S1, Supporting Information). Figure 1e schematically depicts the critical supersaturation and stable saturation concentration of C_10_‐DNTT dissolved in an organic solvent as a function of the solution temperature. From the solubility curve, we know that a dilute yet stable C_10_‐DNTT solution can reach the supersaturation state by increasing the concentration and/or decreasing the temperature. The nucleation process would only occur in the supersaturated zone to form a crystal.^49^ The supersaturation region in Figure 1e consists of two subzones, one is a metastable zone between the solubility and critical supersaturation curves, and the other one is the labile zone where the homogeneous‐nucleation can spontaneously occur.^49^ Lamer and Dinegar have developed a classical nucleation theory in conjunction with modern phase transition mechanisms, in which the metastable zone is further divided into two subzones, namely, the heterogeneous nucleation region and diffusion region.^50^ As labeled in Figure 1e (black vertical dash line), fixing at a constant deposition temperature allows the production of a tiny minority of nuclei (*I* ≈ 0) when the supersaturation of solute is located in the heterogeneous self‐nucleation region (stage B). Furthermore, the self‐nucleation can lead to partial relief of supersaturation, leaving enough and opportune solute to produce facile crystal growth in the diffusion range (stage C). As a result, a low C_10_‐DNTT concentration and a low deposition temperature (50 °C) were employed in our experiments to suppress the unwanted homogeneous nucleation caused by fast increasing *S* due to rapid evaporation of the solvent near the meniscus line. Furthermore, the low processing temperature significantly reduces thermal‐crack defects. To the best of our knowledge, this is the first report of 2D organic monolayer sheets with suppressed thermal‐crack defects and reasonably good aligned orientation developed by a single blade shearing over a large area.

In the USS deposition, a low‐temperature of 50 °C, 0.1 mg mL^−1^ C_10_‐DNTT in tetralin solvent and an ultraslow shearing speed of 1 µm s^−1^ were chosen for the monolayer fabrication after an optimization process. As shown in the polarized optical microscope (POM) image (**Figure** **2**a), uniform and compact C_10_‐DNTT monolayer crystals with large coverage of several centimeter square were obtained. The morphology and thickness of the obtained monolayer are characterized by an atomic force microscope (AFM) and the results are shown in Figure S2 (Supporting Information). More AFM profiles were taken at different crystal domains of the monolayer film also showed very similar surface morphologies and thickness steps (Figure S3, Supporting Information). It can be seen that the crystal surface is atomically smooth (root‐mean‐square (RMS) roughness is 0.4 nm) without any thermal cracks. The measured thickness is around 3.82 nm, corresponding to the height of a single C_10_‐DNTT molecule along the *c*‐axis direction. The single‐layer property was further confirmed by X‐ray reflectivity (XRR) through fitting the profile by an XRD software, shown in Figure S4 (Supporting Information). The fitted thickness of 3.90 nm matches well with the length of C_10_‐DNTT crystals along the *c*‐axis direction.

**Figure 2:**
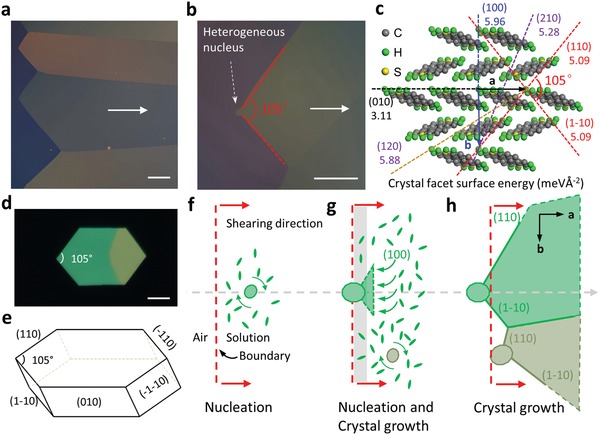
C_10_‐DNTT crystal monolayer obtained with USS method and its crystal growth mechanism. a) Polarized optical microscopy (POM) images of the C_10_‐DNTNT monolayer crystals. The white arrow shows the shearing direction. b) The crystal film with a clear nucleation head demonstrates the formation of crystals by the heterogeneous nucleation process. c) Surface energies (γ) of C_10_‐DNTT low‐index planes obtained from DFT calculations. The molecular side‐chains are omitted for clarity. d) Crystal shape formed without solution movement. The scale bar represents 40 µm. e) Schematic for equilibrium crystal shape of C_10_‐DNTT. f–h) Schematic for crystal growth processes in USS method. Red dash lines indicate the meniscus line (air/solution boundary). The ellipses and herringbones represent the nuclei and C_10_‐DNTT molecules in the solution, respectively. The small nucleus as indicated with a small ellipse in (f) indicates its initial status, which rotates freely (as indicated by rotating arrows) and grows with the shearing process. The light gray region in (g) displays the region with scarce molecules. h) Crystals developed from two nuclei merge together and the crystal boundary forms.

The domains of the crystal deposited by the USS method exhibit a special triangular shape at the beginning seed of the crystals. In a high magnification of POM image displayed in Figure 2b, one notices that the triangular shape was originated from a tiny heterogeneous nucleus. The angle is around 105°, which is in line with the diagonal angle of the crystallographic *a*–*b* unit plane as shown in Figure 2c. In addition, the bisection line of the diagonal angle is parallel with the shearing direction, which indicates the shearing direction is close to the *a*‐axis of the C_10_‐DNTT crystal. Obviously, the edge line of the triangular shape should align with the (110) or (1–10) planes. The triangular crystal heads were also observed with different angles that were formed by other crystal facets, as shown in Figure S5 (Supporting Information). An approach based on density functional theory (DFT) was employed to investigate the growth mechanism of these shaped crystals by calculating the surface energies of C_10_‐DNTT crystals, which usually play a crucial role in the growth of crystals through the Wulff's law.^49^, ^51^, ^52^ Since the surfaces with higher Miller indices are difficult to observe due to their instability, only the low‐index facets with Miller indices up to two have been selected for the DFT calculations. The calculated surface energies (meV Å^−2^) are depicted in Figure 2c. As a comparison, the thermodynamics of the growth process has been first investigated under the condition when the blade has no movement, in which the attaching molecules can approach from all directions and stick to an initial nucleus center and an equilibrium crystal shape (ECS) with Wulff's construction was observed in experiment (Figure 2d). In this case, according to Wulff's theorem, the normal distance from the crystal center to an external facet is proportional to its surface energy.^52^ This could explain that the facet (100) is absent from the ECS because of the highest surface energy of 5.96 meV Å^−1^ among our calculated facets, as shown in Figure 2e. On the contrary, facets with lower surface energies (e.g., (010) and (110)) could steadily appear in the end.

In the USS method, due to the movement of the shearing blade, the crystal growth condition is different and results in the elongated crystal domains with a preferential orientation. The mechanism for the formation of these preferentially elongated crystals is proposed based on the modified Wulff model by including the kinetic effect of the shearing process, as illustrated in Figure 2f–h. The growth mechanism could be explained as follows. At the very beginning of the USS, a small ordered molecular cluster (also referred to as the initial nucleus) forms near the meniscus line (air‐solution boundary) through a heterogeneous nucleation process (Figure 2f). Similar to the no blade movement process, this initial process can be approximated as a near‐equilibrium process due to the small initial nucleus, and it could modulate its in‐plane orientation by rotating under the effect of shearing movement.^49^ To satisfy the exposure of low surface energy (110) or (1–10) planes, the shearing force allows the initial nucleus to align its (100) surface normal along the shearing direction (Figure 2g). The kinetic molecule transportation is enhanced along the shearing direction, and thus the stacking probability along the shearing direction is much higher than those in other directions. This higher stacking probability could satisfy the fast growth velocity requirement of the (100) face resulted from the largest surface energy. The resultant balance between the molecule adsorption and supply promotes the sequentially continuous crystal growth by solution‐phase van der Waals epitaxy in our USS method. Since the formed nucleus has a limited size, the crystal also expands towards two side directions besides along the (100) surface normal. As shown in Figure 2h, the stable (110) and (1–10) faces appear in the end. In a few other samples, (210) and (2–10) faces (with higher surface energies) are observed as well (Figure S5, Supporting Information). Furthermore, in the USS process, there would be other nuclei formed nearby and if their (100) surfaces normal finally align precisely along the shearing direction, the crystal domains will merge together to form a larger perfect single crystal. Otherwise, the crystals developing from these two nuclei will form grain boundaries (Figure 2h), and these two deposition modes would occur together on the same substrate. In short, the growth pattern of the C_10_‐DNTT crystal in our USS method can be attributed to a synergistic effect between thermodynamics and kinetics of growth, whereby the presence of a free surface at the beginning is attributed to the lower surface energy while the preferential molecular orientation along shearing direction is caused by the anisotropic sticking probability and thus growth velocity.

We further confirmed the occurrence of the heterogeneous nucleation by modifying the supersaturation ratio through varying the solute concentration. If we increase the initial concentration of the C_10_‐DNTT solution from 0.1 to 0.5 mg mL^−1^, uneven but compact thick films with small grains and bright spot‐defects can be accessible (Figure S7a, Supporting Information). The bright spot‐defects are believed to be caused by an easier concentration fluctuation involving a partial homogeneous nucleation process at relatively high concentration, which are missing in the USS method with a lower solute concentration. The surface morphology of films fabricated under a fast shearing speed (10 µm s^−1^) was compared in Figure S7b in the Supporting Information, the abundant large gaps are induced by the unbalanced diffusion rate between the evaporation of the solvent and the consumption of C_10_‐DNTT molecules.^15^, ^53^, ^54^ The detailed discussion about the correlation between the solvent evaporation and the shearing speed can be found in ref. 53. At high deposition temperature around 90 °C, the concentration of solution near the meniscus line drastically increased due to the rapid evaporation of the solvent. The whole deposition system falls into the labile‐zone discussed in Figure 1e; therefore, the random and spontaneous nucleation appearing at the very beginning of the deposition (Figure S7c, Supporting Information) is detrimental to the formation of large size single‐crystalline domains. High temperature also breeds many thermal cracks in the organic film. These cracks are aligned with *a*‐axis, i.e., the (010) planes of the C_10_‐DNTT crystals,^55^ which has the lowest surface energy of 3.11 meV Å^−2^. From the Kelvin probe force microscopy (KPFM) measurement near the crack region shown in Figure S8 in the Supporting Information, the crack shows a downshifting of the Fermi level (≈0.03 eV) which we believe to be due to the hole trapping properties of the cracks. It is also important to mention that even when the grain boundary cannot be detected in the topography profile (Figure S8c, Supporting Information), they could be clearly identified by the corresponding potential profile (Figure S8d, Supporting Information). These trap states would degrade the carrier transportation in the OFETs to a certain extent.^56^, ^57^, ^58^

The high quality of 2D crystals grown by the USS method under 50 °C and 0.1 mg mL^−1^ was further verified by the X‐ray diffraction (XRD) technique. **Figure** **3**a shows a 2D diffraction image of single‐layered C_10_‐DNTT crystals on the silicon substrate measured by grazing‐incidence wide‐angle X‐ray scattering (GIWAXS). Although the C_10_‐DNTT crystals are only monolayer thick, bright spots along the in‐plane direction can still be detected. No spots were obtained in the *Q_z_* coordinate due to the dissatisfaction of Bragg diffraction conditions for the monolayers. This is consistent with the general theta/2theta (θ/2θ) XRD spectrum with zero peaks, as shown in Figure 3b. In order to confirm the existence of the preferential molecule orientation observed by the POM in Figure 2b, the in‐plane phi‐scan (φ‐scan) measurement for detecting (020) planes of the C_10_‐DNTT monolayer was implemented with 2θ settled at 22.64° via rotating the sample by a circle. From the φ‐scan result (Figure 3c), we can see that the two strongest and needle‐like peaks are located at 14.5° and 194.5°, which verifies the shearing direction is roughly aligned with the *a*‐axis of C_10_‐DNTT again with only about 3° misalignment. The incident direction of the X‐ray is parallel to the shearing direction on purpose during the measurement.

**Figure 3:**
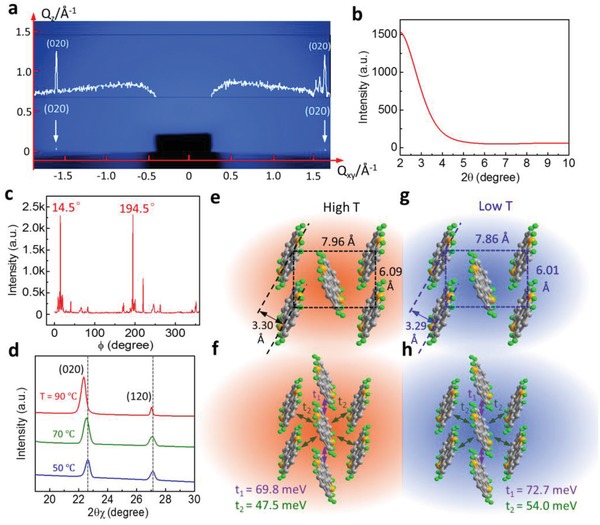
Characterizations of the crystals fabricated by the USS method. a) Grazing‐incidence wide‐angle X‐ray scattering (GIWAXS) image of C_10_‐DNTT monolayer on the wafer substrate. The incident angle is kept at 0.15° and the exposure time is 100 s. The white profile shows the correspondingly converted spot signals. b) General theta/2theta XRD result of the C_10_‐DNTT monolayer. c) In‐plane XRD phi‐scan result of the (020) planes of the C_10_‐DNTT monolayer crystal. The grazing incident angle is 0.15° and the associated angle of the (020) planes is 22.64°. d) In‐plane XRD profile of detecting (020) planes. e,g) Molecular packing structure of C_10_‐DNTT crystals fabricated at high temperature (90 °C) and low temperature (50 °C), respectively. f,h) The corresponding calculated transfer integrals of C_10_‐DNTT crystals with the structure in e,g) through the Amsterdam Density Functional (ADF) program.

Moreover, a noticeable increase in the peak position of the (020) planes to a higher 2θ value (2θ shifts from 22.37° to 22.64°) happens in the in‐plane GIWAXS structure measurements (Figure 3d) when the C_10_‐DNTT films deposition temperature decreases from 90 to 50 °C. It indicates a tighter molecular packing for the C_10_‐DNTT molecules at the lower temperature. This more compressed lattice structure of C_10_‐DNTT crystals is likely from the shrink of intermolecular distance in the low‐temperature (*a* = 6.01 Å, *b* = 7.86 Å) process than that of in high temperature (*a* = 6.09 Å, *b* = 7.96 Å) as indicated in Figure 3e,g. The expanded *a*–*b* cell structure in high‐temperature C_10_‐DNTT crystals would decrease the charge transfer integrals between the molecules from 72.7 to 69.8 meV and 54.0 to 47.5 meV in the t_1_ and t_2_ direction as shown in Figure 3f,h. Based on the comparisons, the low‐temperature processing C_10_‐DNTT crystals tend to achieve higher mobility in OFETs due to the larger transfer integrals. After confirming the crystallinity and the atomically flat surface of the C_10_‐DNTT monolayers, we measured field‐effect carrier mobility of OFETs based on the monolayer crystals developed by the USS approach with thermally evaporated Au and 2,3,5,6‐tetrafluoro‐7,7,8,8‐tetracyanoquinodimethane (F_4_‐TCNQ) as the source‐drain contacts. The mobility of the monolayer OFET is only around 2.81 cm^2^ V^−1^ s^−1^ (*a*‐axis, the higher mobility direction of C_10_‐DNTT)^55^ and a typical transfer curve is shown in Figure S9 (Supporting Information). When we further increased the deposition temperature to 75 °C, the carrier mobility was further reduced to 0.5 cm^2^ V^−1^ s^−1^ which is attributed to a high density of thermal induced cracks and decreases of the transfer integral. Although the correlation between the film thickness, crack density, crack width, and thermal stress is beyond the scope of the current study, it is definitely worth more in‐depth investigations in the future. Other factors causing the relatively low effective mobility of the monolayer devices include the degradation of crystals in the contact region during the thermal evaporation of metal^26^ or the thickness‐induced mobility degradation caused by the Coulomb scattering phenomenon.^59^, ^60^ It can be partially disentangled by depositing an encapsulation layer such as PMMA or parylene on the atomically thin channel.^55^ Although the mobility has been improved by this way, the encapsulation layer also brings the limitation to 2D semiconductors for the applications of chemical or biochemical sensors, where direct exposure and contact between stimulators and the active channels are required. To address these shortcomings, we thermally evaporate another ultrathin C_10_‐DNTT layer (10 nm) on top of the highly crystalline C_10_‐DNTT monolayer before the deposition of the electrodes. Other than serving as the channel layer, the USS grown 2D film also serves as the growth templates. Compared with the films with a similar thickness (14 nm) deposited directly by thermal evaporation (TE), the hybrid thermal‐evaporation (HTE) films also showed a high crystallinity with the same molecular packing as the bottom templating layer with much better electrical performance. Janneck et al. and Wang et al. have applied similar hybrid layers on another material, 2,7‐dioctyl[1]benzothieno[3,2‐b][1]benzothiophene (C_8_‐BTBT) for the meniscus‐guide coated and spin‐coated OFETs, respectively.^24^, ^61^ Compared with the current C_10_‐DNTT monolayer, their C_8_‐BTBT under ribbon structure has a larger gap for the epitaxy layer to fill up hence the required thicknesses of the epitaxy layer is also higher. Moreover, the exact molecular orientation and crystallinity of the upper thermal evaporated layer remain unknown. Here we will take a few steps further to investigate the correlation between the bottom C_10_‐DNTT monolayer and the upper thermal evaporated thin layer.

Schematic illustration of the differences between the random growth by TE and templating vertical quasi‐epitaxial growth by the HTE method is depicted in **Figure** **4**a. From the scanning electron microscopy (SEM) image in Figure 4b, the TE films are strictly polycrystalline with micrometer‐sized domains. Each domain contains many tall crystal whiskers that are formed during the thermal deposition with the substrate temperature of 50 °C. These whisker‐like lamellas have been reported to be parallel with the direction of *a*‐axis, i.e., [100].^62^ Consequently, we can judge the crystal orientation of each crystal domain using these lamellas as markers. In the case of HTE films, the thermally evaporated compact film with the same lamellas on the surface and almost all the lamellas are parallel to each other within each crystal domain (Figure 4c). An ultraslow evaporation rate of 0.1 Å s^−1^ was used to avoid structural disorder.^63^ The low evaporation rate allows the subsequent gas molecules to access an energy‐favorable site leading to well‐ordered growth with minimum structure disorder.^64^

**Figure 4:**
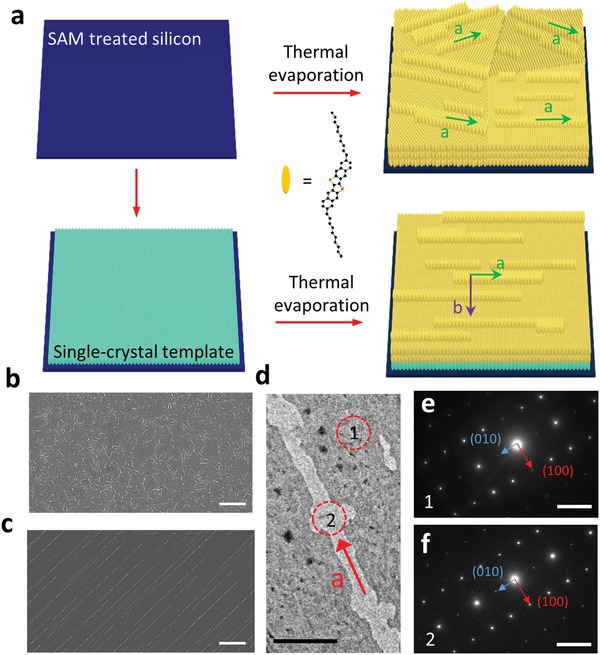
Monolayer single crystal domains as templates for depositing another upper layer by thermal evaporation. a) Schematic illustration of vertical quasi‐epitaxial growth. b) SEM image of the C_10_‐DNTT films fabricated on the pure substrate. c) SEM image of the C_10_‐DNTT crystalline film with a monolayer crystal template. The scale bars in (b) and (c) are 1 µm. d) A TEM micrograph of the hybrid C_10_‐DNTT film and e,f) are corresponding SAED patterns for the zone (1) and (2) in (d). The scale bar in (d) is 1 µm and in (e) and (f) are 2 1/nm.

The effect of the template on the thermally evaporated thin film was further confirmed by the transmission electron microscopy (TEM) and select area electron diffraction (SAED) analytical techniques. In the case of HTE film, as shown in Figure 4d–f, similar diffraction patterns were observed at selected regions 1 and 2, confirming the crystalline nature of the upper layers. The highly ordered upper layers were realized by the interfacial interaction between the template and upper layers,^65^ which promotes the monocrystalline structure coherence of the hybrid films with a perfect continuity of molecular arrangements. In order to further verify the monocrystalline property within one particular domain in the organic thin film, we captured the SAED patterns at different positions of the same domain, and very similar SAED patterns can be observed (Figure S10, Supporting Information). In the case of pure TE films, the amorphous C_10_‐DNT can also be confirmed by its multiringed SAED pattern, as shown in Figure S11 (Supporting Information). The difference between the TE and HTE films was further investigated by the GIWAXS on a larger scale (1 cm × 1 cm) samples. As displayed in **Figure** **5**a, the diffraction rings present the polycrystal structure of the TE films, which is in agreement with the TEM observation. On the other hand, the hybrid films fabricated by HTE exhibit extremely high crystallinity as single crystals in terms of a single domain but with higher peak intensities due to a thicker film. Several rows of diffraction spots were clearly observed along both in‐plane and out‐of‐plane directions (Figure 5b), which demonstrated that the highly crystalline hybrid films were achieved by the HTE method.

**Figure 5:**
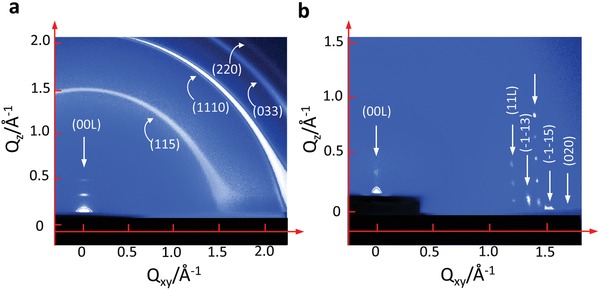
GIWAXS pattern of C_10_‐DNTT thin films. a) C_10_‐DNTT films fabricated by direct thermal evaporation. b) Hybrid C_10_‐DNTT films with solution‐processed and highly crystalized monolayers as templates fabricated by thermal evaporation. The incident angle is kept at 0.15° and the exposure time is 600 s.

The performance of the transistors based on HTE films is compared with the reference samples based on 14 nm thermal evaporated C_10_‐DNTT. The channel length and width of transistors were 76 and 500 µm, respectively. Before the electrical measurement, all devices were patterned by mechanical scratches to avoid the fringe current.^66^, ^67^ All the OFETs were characterized in a nitrogen glove box at room temperature. As transfer curves shown in **Figure** **6**a, OFETs with HTE C_10_‐DNTT films as active layers exhibit excellent electrical performances with an averaged value of 14.7 cm^2^ V^−1^ s^−1^ based on over 45 devices. The highest carrier mobility is 16.0 cm^2^ V^−1^ s^−1^, and the relatively small standard deviation is 0.66 cm^2^ V^−1^ s^−1^. Figure 6c illustrates a typical output curve of the HTE devices. The statistic histogram of mobility of the measured devices is summarized in Figure 6e. Comparing with the performance of the referenced TE samples (Figure 6b,d), a 100% increase of average mobility can be achieved (7.34 to 14.65 cm^2^ V^−1^ s^−1^). No kinks are observed in the square‐root‐plot of drain‐source current, which indicates the contact quantity is good and the measured mobility is not affected by the contacts. The linearity of the transfer curves was further confirmed by the negligible mobility dependence on the gate voltage in Figure 6f. One possible cause of the improved carrier mobility in HTE devices is due to the more efficient carrier injection at the contacts of the HTE. The upper C_10_‐DNTT layer can protect the solution processed monolayer during the deposition of metal. Lastly, the ambient stability of the devices is examined. Figure S12 (Supporting Information) shows the air‐stability of HTE and TE devices after keeping under ambient condition with high humidity of 60% for 45 days. The HTE transistors demonstrates better shelf‐life stability with only a slight drop of 7% in carrier mobility after exposure to air for 45 days while the TE devices decreased substantially by 26% in the same period.

**Figure 6:**
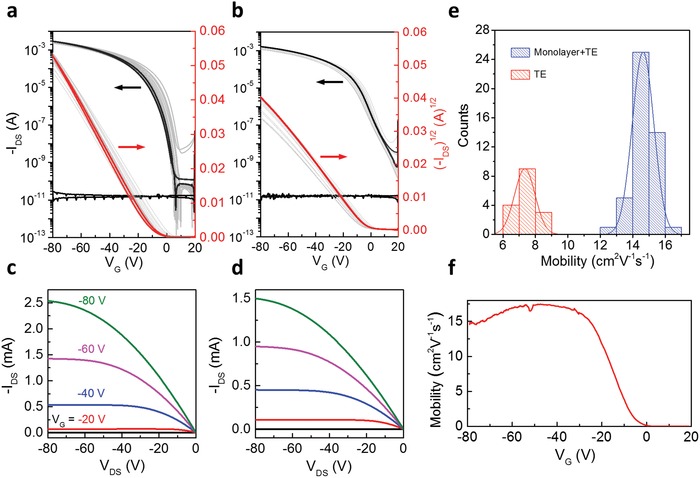
Transistor characteristics of OFETs based on C_10_‐DNTT films. a) Transfer curves of hybrid films with highly crystalized monolayer as thermal evaporation templates (HTE). b) Transfer curves of films deposited simply by thermal evaporation (TE). c,d) One of the typical output curves of HTE and TE devices, respectively. e) Statistic histogram of the carrier mobility. f) The µ‐V_G_ dependency of the devices based on HTE C_10_‐DNTT films. Channel width (*W*) to length (*L*) of OFETs is 6.6 (500/76 µm).

In summary, we fabricated a highly crystalline organic monolayer with large‐area uniformity by an ultraslow shearing method which significantly reduces the number of nuclei. The growth pattern of the monolayer shows a kinetic Wulff's construction with a preferential molecular orientation supported by theoretical calculations of surface energies. With the obtained 2D crystals acting as templates, we further integrate it with a thermally evaporated thin film to form a hybrid layer OFET channel. Due to the vertical quasi‐epitaxial growth, the evaporated upper layer exhibits the same crystal orientation with the long‐ranged ordered crystalline. The field‐effect transistors based on the hybrid‐deposited organic semiconducting films exhibited outstanding carrier mobility up to 16.0 cm^2^ V^−1^ s^−1^ and good shelf‐life stability. We confirmed the highly crystalline upper layer plays an important role in the improvement of the charge transport in the bottom channel as well as the ambient stability. This work establishes a simple yet effective deposition approach to regulate the growth direction of the thermal evaporated organic thin film. The hybrid processing method proposed here is ideally suited for the fabrication of high‐performance, flexible, and low‐cost electronics on a large scale.

## Experimental Section

Experimental details are given in the Supporting Information.

## Conflict of Interest

The authors declare no conflict of interest.

## Supporting information

Supplementary

Supplementary
