# Supplementary material for: Field‐Effect Transistors Based on 2D Organic Semiconductors Developed by a Hybrid Deposition Method
Source: Adv Sci (Weinh). 2019 Aug 1;6(19):1900775. doi: 10.1002/advs.201900775 (PMC6774035; doi:10.1002/advs.201900775)
Supplement: Supplementary file 1 — Supplementary [file ADVS-6-1900775-s002.pdf]

Copyright WILEY-VCH Verlag GmbH & Co. KGaA, 69469 Weinheim, Germany, 2019.

ADVANCED SCIENCE Open Access

Supporting Information

for *Adv. Sci.,* DOI: 10.1002/advs.201900775

Field-Effect Transistors Based on 2D Organic Semiconductors Developed by a Hybrid Deposition Method

Zhiwen Zhou, Qisheng Wu, Sijia Wang, Yu-Ting Huang, Hua Guo, Shien-Ping Feng, and Paddy Kwok Leung Chan**

Copyright WILEY-VCH Verlag GmbH & Co. KGaA, 69469 Weinheim, Germany, 2019.

Supporting Information

Field-Effect Transistors Based on Two-Dimensional Organic Semiconductors Developed by a Hybrid Deposition Method

Zhiwen Zhou[1], Qisheng Wu[2], Sijia Wang[1], Yu-Ting Huang[1], Hua Guo[2], Shien-Ping Feng[1] and Paddy Kwok Leung Chan[1*]*

[1]Department of Mechanical Engineering, The University of Hong Kong, Pok Fu Lam Road, Hong Kong
[2]Department of Chemistry and Chemical Biology, The University of New Mexico, Albuquerque, NM 87131, USA

*E-mail: pklc@hku.hk
*E-mail: hpfeng@hku.hk

EXPERIMENTAL METHODS

Materials: Silicon substrates with 300 nm thermally grown oxide layers as dielectric were purchased from University Wafer Inc. High purity of C_{10}-DNTT (99.5%) organic semiconductor was provided by Prof. Kazuo Takimiya and Dr. Yuichi Sadamitsu from Nippon Kayaku Company. The solvents and SAMs were obtained from Sigma-Aldrich. All materials were used as received.

Ultra-Slow Shearing Process for Monolayer Crystals Deposition: The blade-shearing system was built by ourselves and for the details, you can refer to our previous study. In order to prevent the deposition of organic crystals on the surface of the shearing blade, it was treated with octadecyltrichlorosilane (OTS) to form a hydrophobic surface. On the other hand, the silicon substrate was treated by another self-assemble monolayer trichloro(phenethyl)silane (PTS) to increase the substrate surface energy and make it hydrophilic. The PTS treatment can enhance the crystallinity of deposited organic films. During the monolayer deposition process, the optimized substrate and blade temperature was 50-55℃ for the tetralin solvent in order to avoid the formation of thermal cracks. Due to the low solubility of C_{10}-DNTT, the monolayer crystals were deposited by a small concentration of 0.1 mg/ml in tetralin solution. To balance the evaporation rate of solvent and sink rate of C_{10}-DNTT molecules, an ultraslow shearing speed of 1 μm/s was adopted. The deposition temperature can be further lowered to near room temperature (~35℃) by using m-xylene as the solvent.

Crystal Characterizations: Crystal orientation and uniformity were roughly characterized by a polarized optical microscope (Nikon Eclipse LV100N). The surface morphology and thickness of crystals were measured by the Bruker Multimode 8 under ambient environment. The SCM-PIT-V1 (Pt-Ir, frequency ~75 kHz, Bruker) tip was used for the KPFM measurement with

tapping mode in ambient conditions. The work function of the tip was calibrated by standard Kelvin probe sample (PFKPFM-SMPL, Bruker) with alumimum and gold patterns on a silicon substrate. The 2D-GIWAXS experiments were carried out by a *Rigaku SmartLab X-Ray* diffractometer with a latest 2D camera detector (*HyPix-3000*), a special optical attachment (*CBO-f*) and micro-sized collimators. The high-energy X-ray radiation source was generated by a Copper target with highest energy power of 9 kW. A knife edge and scattering shield were used for suppressing the effect of air scattering caused by an incident X-ray beam. For this analysis, the incident angle of the X-ray was set to 0.15-0.20° (ω = 0.15°-0.2°) and clear diffraction images were obtained with suitable exposure time. The out-of-plane, in-plane XRD and XRR results were based on the same equipment. The XRR profile was analyzed using a multilayered stack model, in which each C_{10}-DNTT molecule was divided into two aliphatic C10 and one aromatic DNTT slabs. By this model, a well-fitted result of experimental XRR profile was obtained by using *Rigaku GlobalFit* software. The SEM images were captured by LEO 1530 scanning electron microscope. Transmission electron microscopy (TEM) and SAED experiments were performed on an FEI Tecnai G2 20 S-TWIN with a high resolution of 2 Å.

OFETs Fabrication and Measurements: Silicon substrate was cleaned by standard substrate cleaning procedure including oxygen plasma treatment and PTS SAM modification which has been reported before. For C_{10}-DNTT organic film fabrication, the monolayer single-crystalline templates were obtained by the USS method. Based on the templates, another same ultrathin layer was deposited on the top of the template by thermal evaporation. The reference film was fabricated by pure thermal evaporation. The parameters of the thermal evaporation process were kept the same in the above two cases with a vacuum of 2×10^{-6} Torr and the deposition rate of 0.1 Å/s. The substrate temperature was 50℃ during the thermal deposition. After the organic

semiconductor deposition, we utilized 60 nm gold as the source/drain electrodes with 4 nm F_4-TCNQ charge injection layer deposited by thermal evaporation. The channel length and width of transistors were 76 and 500μm, respectively. All the electrical characterizations were performed under a nitrogen environment and the detailed testing procedures can be found in our previous work. Before the electrical measurements, all the devices were annealed at 50 °C for 12 hours in the glove box.

Details of DFT calculations

The theoretical calculations were executed by using density functional theory (DFT) methods using projector augmented wave (PAW) pseudopotentials as implemented in Vienna ab initio simulation (VASP) 5.4 package.[1] Perdew-Burke-Ernzerhof parametrization of the generalized gradient approximation (PBE-GGA) was employed.[2] The van der Waals (vdW) interactions were described with the Grimme scheme of DFT-D2 for semiempirical correction.[3] The plane-wave cutoff energy was set to 520 eV and vacuum space larger than 12 Å were simulated to minimize the interaction between nearby layers. All systems were completely geometrically optimized until the energy and force met the criteria of 10^{-5} eV and 0.02 eV/Å, respectively. To prove the accuracy and effectiveness of our DFT methods, we made the comparisons of calculated formation energies and surface energies between our results and Louie's work[4] based on the same pentacene crystal structure below. The transfer integrals were evaluated using GGA: PW-91 TZP package in Amsterdam Density Functional (ADF) program.[5]

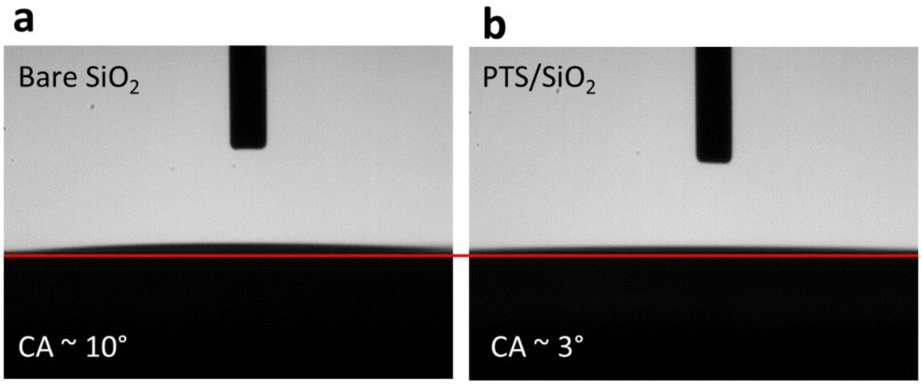

Figure S1. Contact angle θ of tetralin solvent on bare SiO_2 and PTS treated SiO_2 surfaces. The volume of the tetralin used in the measurements is around 3 μL.

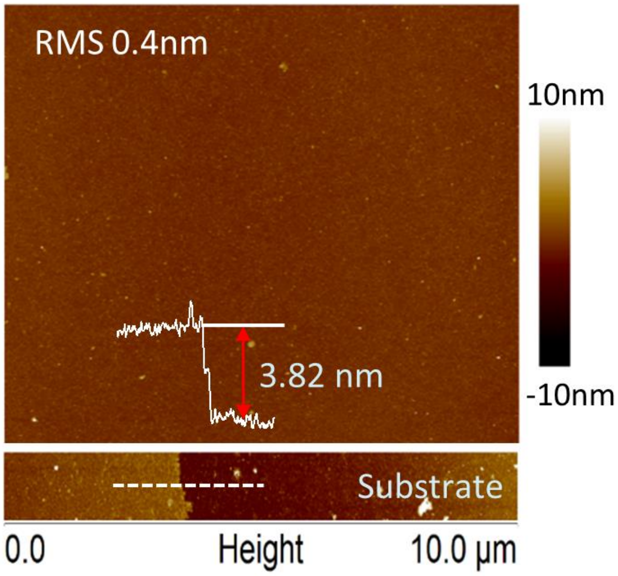

Figure S2. Morphology characterizations of C_{10}-DNTT monolayers. AFM snapshots of surface morphology and thickness of the crack-free monolayer sample.

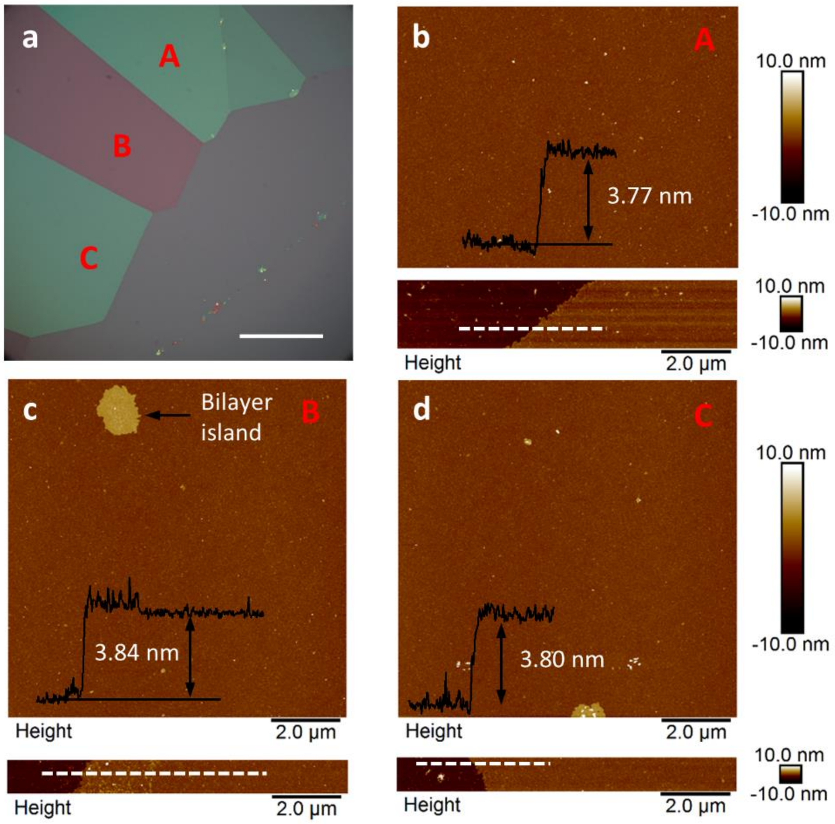

Figure S3. AFM profiles were taken in different grain domains of the monolayer crystals. (a) POM image of C10-DNTT monolayer crystals. Grain A, B, C have different in-plane molecular arrangements. The scale bar is 400μm. (b)- (d) The morphology and thickness measured at the edge of grain A, B and C, respectively. They showed similar surface topography and film thickness.

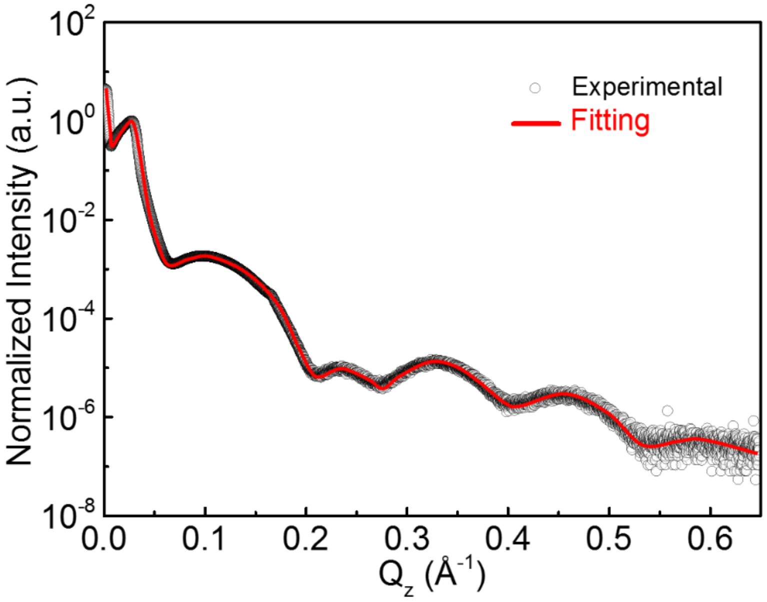

Figure S4. Characterization of C_{10}-DNTT monolayers by X-ray reflectivity (XRR) technique. The experimental result of XRR measurement and its fitting result. The fitted thickness is around 3.90 nm, which is consistent with the result of AFM test.

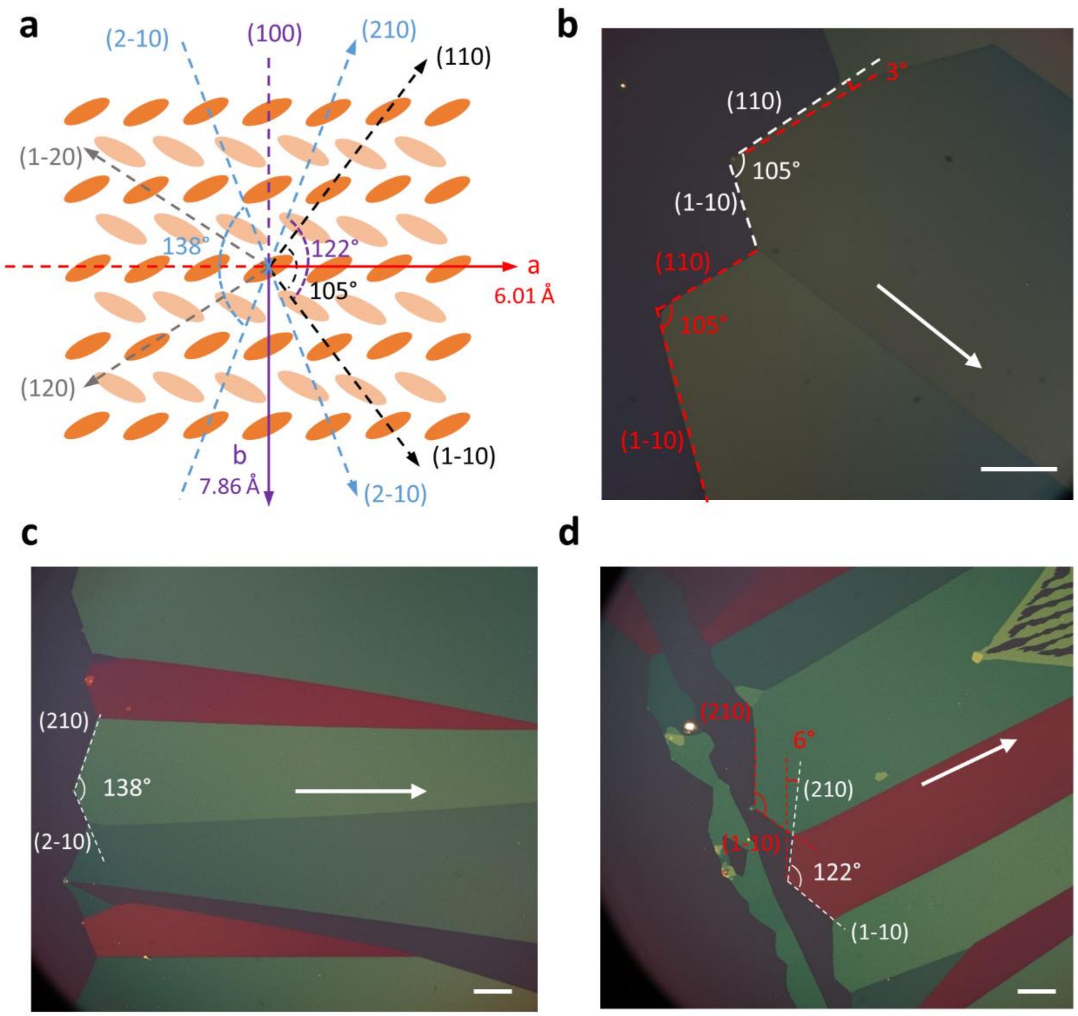

Figure S5. Schematic a-b plane crystal structure of C_{10}-DNTT and crystals deposited by USS method. (a) Schematic diagram of herringbone packing mode with marked low-index planes and some of their intersection angles. (b) Crystal domains with clear triangular heads and the triangles are around 105°. The two adjacent single crystal domains have about 3° molecular orientation mismatch, i.e., the grain boundary of them is 3°. (c) The crystal domain with triangular heads of 138°. (d) The crystal domains with triangular heads of 122° and the grain

boundary of them is around 6°. The white arrow in all POM images shows the shearing direction and the scale bars are 200 µm.

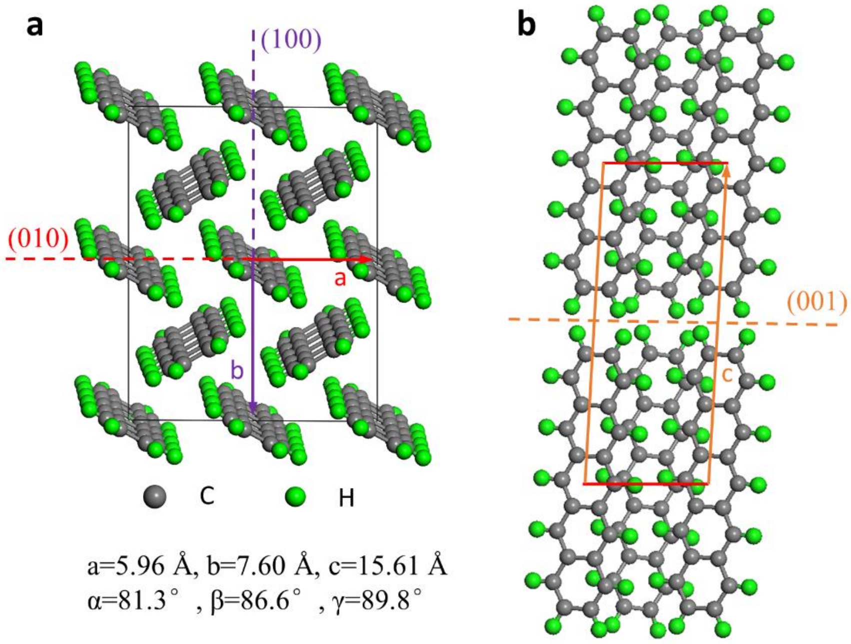

Figure S6. Surface energy (γ) calculations of pentacene low-index planes by DFT method. (a) Schematic view of a single a-b layer of pentacene and its unit cell parameters that are the same as those used in Louie's work. (b) Side view of pentacene.

Table S1. Comparisons of calculated formation energy and surface energy between our DFT calculations and Louie's work based on the same pentacene crystal structure.

	**Formation energy, E_{form}/cell (eV)**		**Surface energy, γ (meV/ Å^2)**	
	Louie's work	This work*	Louie's work	This work*
(100)	0.75	0.795	6.4	6.68
(010)	0.45	0.442	4.8	4.75

(001)	0.15	0.201	3.1	4.44

*Notes: Our DFT-D2 results are generally in accord with that in Louie's work in the reference, during which van der Waals interactions have not been implemented in DFT codes. It is believed that our DFT-D2 results would be more close to experimental values considering the unavoidable van der Waals interactions in pentacene crystals. Therefore, the surface energy of C_{10}-DNTT crystals is calculated with our DFT-D2 method in this work.

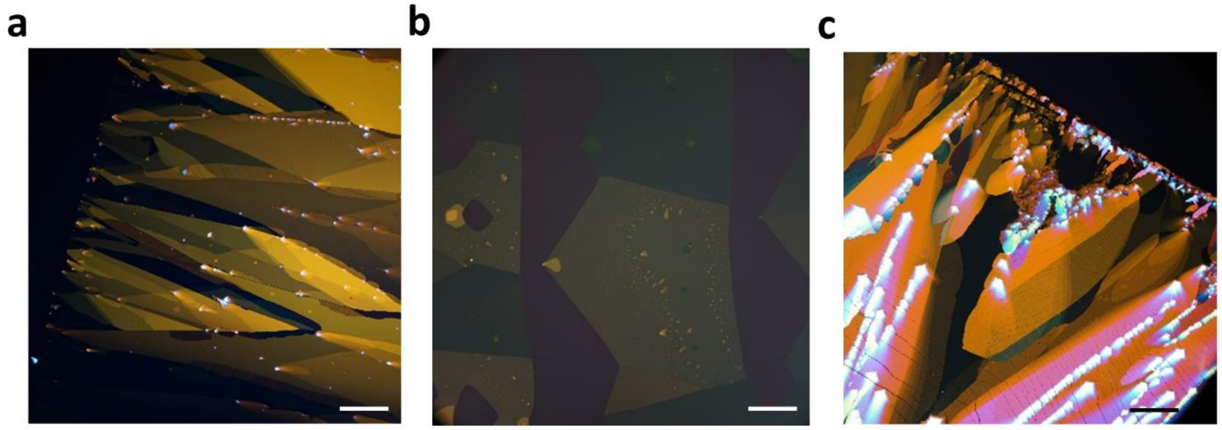

Figure S7. C_{10}-DNTT Crystals deposited by blade-shearing method. (a) Crystal formed at a high solution concentration of 0.5mg/ml (T = 55□, v = 1μm/s). (b) Crystals deposited by a fast shearing speed of 10 μm/s (T = 55□, C = 0.1mg/ml). (c) Crystal formed at a high deposition temperature of 90□ (C = 0.1mg/ml, v = 1μm/s). The scale bar in all the POM images are 200 μm.

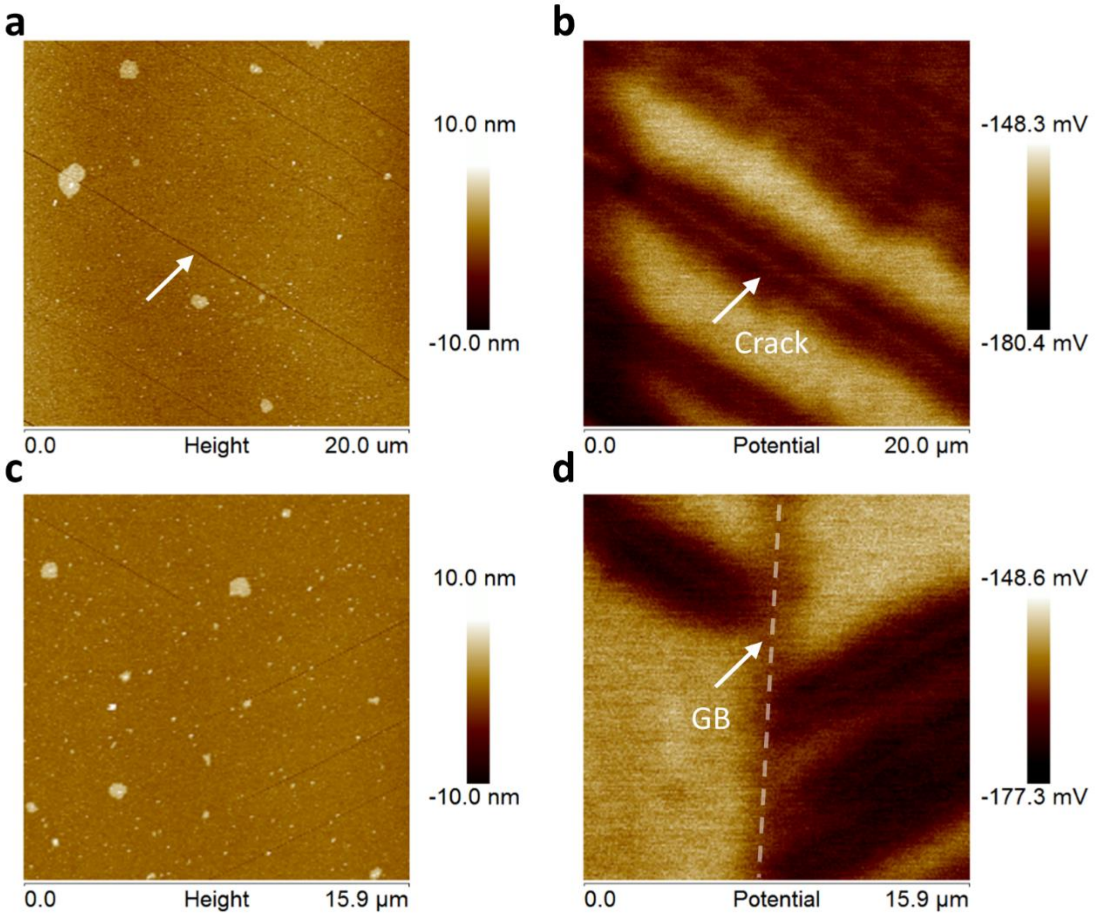

Figure S8. Surface potential measurements of the C_{10}-DNTT ultrathin films with thermal cracks by Kelvin probe force microscopy (KPFM). (a) Topography image of the film within a crystal domain. (c) Topography image of the film crossing two adjacent crystal domains. (b), (d) are corresponding contact potential difference (CPD) profiles of the figure (a) and (c). Interestingly, the grain boundary (GB) is hard to be observed from the topography profile in (c), however, we could easily identify the GB according to the variation of surface potential in the corresponding CPD profile (d).

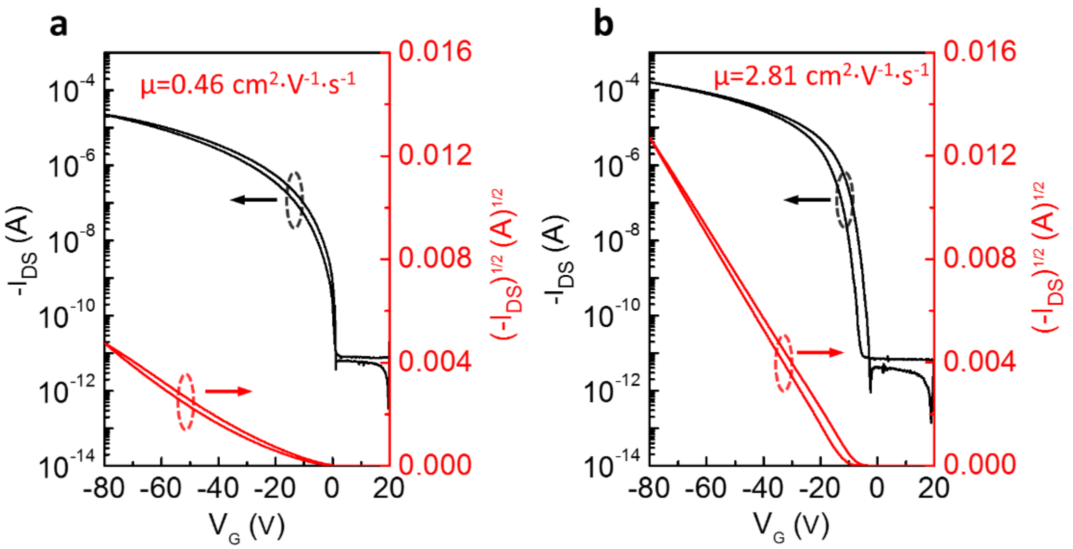

Figure S9. Transfer curves of OFETs based on C_{10}-DNTT monolayers. (a) The monolayer with thermal cracks. (b) The monolayer without thermal cracks. The electrical performances of OFET devices were characterized in the nitrogen glove box. The channel width (W) and length (L) of the OFET devices are 220 and 500μm (W/L ≈ 2.27), respectively.

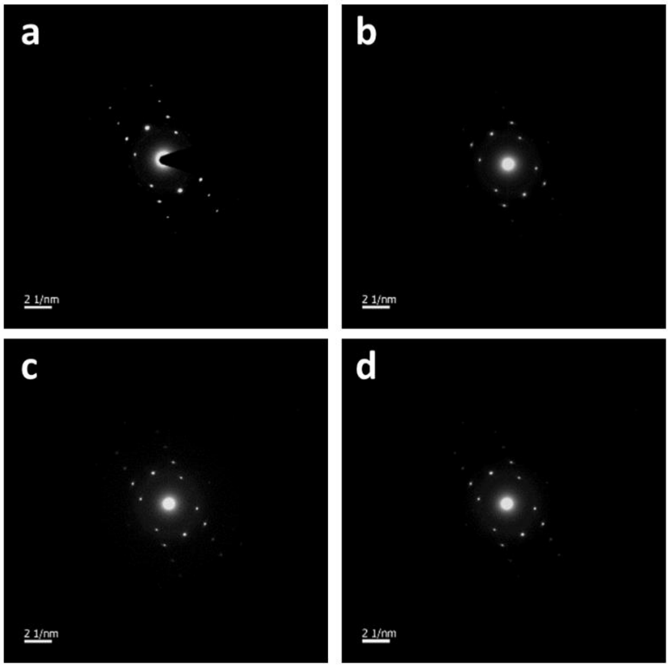

Figure S10. SAED patterns taken for HTE crystalline domains at different positions of the same sample.

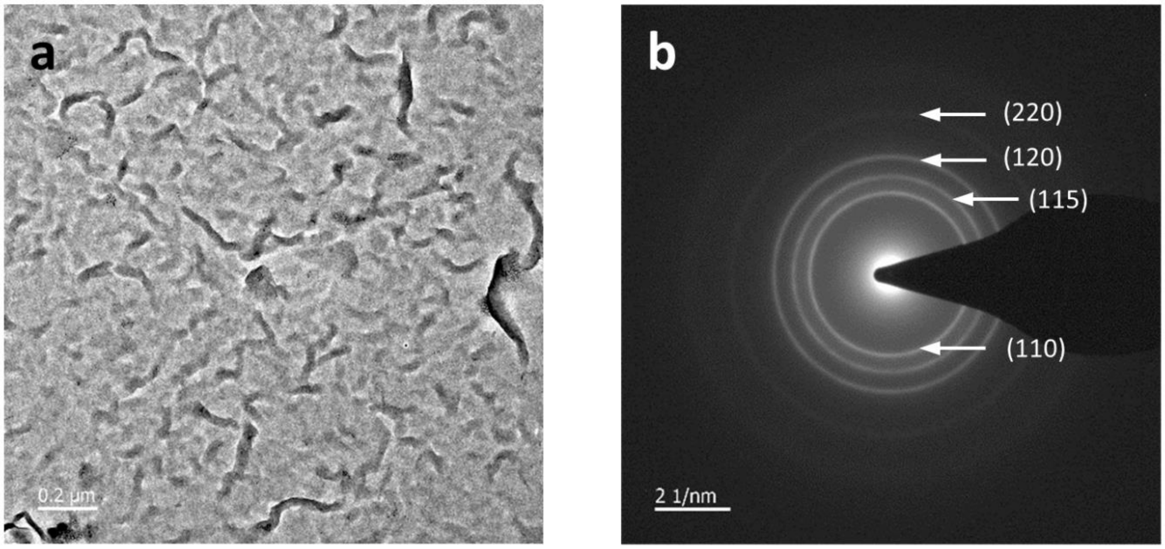

Figure S11. TEM micrograph and corresponding SAED patterns of the film deposited by direct thermal evaporation. (a) Bright field image of polycrystalline C_{10}-DNTT thin film. (b) The corresponding SAED pattern.

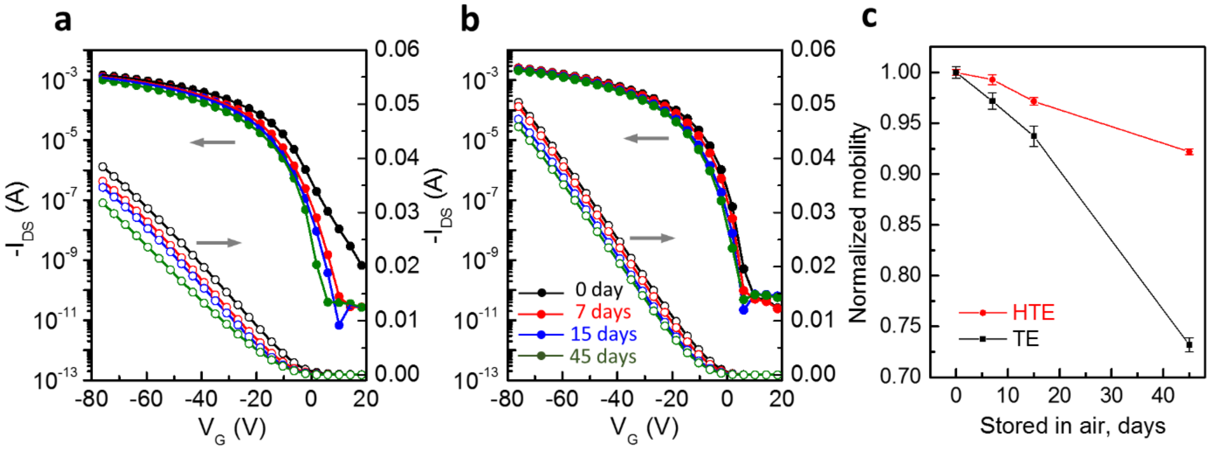

Figure S12. Comparisons of life-stability of C_{10}-DNTT films after exposure to air. (a) Transfer curves and square-root-profiles of I_{DS} based on thermal evaporation (TE) films. (b) Transfer curves and square-root-profiles of I_{DS} based on hybrid thermal evaporation (HTE) films. (c) Comparison of normalized mobility of TE and HTE OFETs stored in air and measured in glove box over the same period.
